# Entomological longitudinal surveys in two contrasted eco-climatic settings in Cameroon reveal a high malaria transmission from *Anopheles funestus* associated with *GSTe2* metabolic resistance

**DOI:** 10.1186/s12879-023-08698-8

**Published:** 2023-10-28

**Authors:** Brice Natchema S. Fonkou, Magellan Tchouakui, Benjamin D. Menze, Leon M. J. Mugenzi, Derrick Fofie, Daniel Nguiffo-Nguete, Lucia Nkengazong, Jeannette Tombi, Charles S. Wondji

**Affiliations:** 1 Medical Entomology Department, Centre for Research in Infectious Diseases (CRID), Yaoundé, Cameroon; 2 Faculty of Sciences, University of Yaoundé I, Yaoundé, Cameroon; 3 Institute of Medical Research and Medicinal Plants Studies, (IMPM, P.O.Box 13033), Yaoundé, Cameroon; 4 Vector Biology Department, Liverpool School of Tropical Medicine, Pembroke Place, Liverpool, L3 5QA UK

**Keywords:** *An. funestus*, Sporozoite infection, Malaria transmission, Entomological inoculation rate, L119F-*GSTe2*, Cameroon

## Abstract

**Background:**

The impact of metabolic resistance to insecticides on malaria transmission remains poorly characterised notably through application of entomological parameters. The lack of resistance markers has been one of the limiting factors preventing a robust assessment of such impact. To this end, the present study sought to investigate how the L119F-*Gste2* metabolic gene influences entomological parameters underpinning mosquitos’ propensity to transmit *Plasmodium* spp.

**Methods:**

Longitudinal studies were carried out in Mibellon and Elende, two different eco-climatic settings in Cameroon and mosquitoes were collected using Human Landing Catch (HLC), Centre for Disease Control Light Trap (CDC-LT) and Pyrethrum Spray Catch (PSC) technics. *Plasmodium* sporozoite parasites were detected by TaqMan and Nested PCR, and blood meal origin by ELISA. The allele-specific PCR (AS-PCR) method was used to genotype the L119F-GSTe2 marker and association with malaria transmission was established by comparing key transmission parameters such as the Entomological Inoculation Rate (EIR) between individuals with different L119F-*GSTe2* genotypes.

**Results:**

*An. funestus* s.l was the predominant malaria vector collected during the entomological survey in both sites (86.6% and 96.4% in Elende and Mibellon, respectively) followed by *An. gambiae* s.l (7.5% and 2.4%, respectively). Sporozoite infection rates were very high in both collection sites (8.7% and 11% in Elende and Mibellon, respectively). *An. funestus* s.s exhibited a very high entomological inoculation rate (EIR) (66 ib/h/month and 792 ib/h/year) and was responsible for 98.6% of all malaria transmission events occurring in both sites. The Human Blood Index was also high in both locations (HBI = 94%). *An. funestus s.s.* mosquitoes with both 119 F/F (RR) and L119F (RS) genotypes had a significantly higher transmission intensity than their susceptible L/L119 (SS) counterparts (IRR = 2.2, 95%CI (1.1–5.2), p = 0.03; IRR = 2.5, 95% CI (1.2–5.8), p = 0.01 respectively).

**Conclusion:**

This study highlights the major role that *An. funestus* s.s plays in malaria transmission in Cameroon with an aggravation from *GSTe2-*based metabolic resistance.

**Supplementary Information:**

The online version contains supplementary material available at 10.1186/s12879-023-08698-8.

## Background

Malaria remains a leading public health concern in Africa, accounting for 29.1% of health facility consultations and 17.2% of deaths in Cameroon [1]. Children under 5 years of age and pregnant women are disproportionately vulnerable; in 2020, hospital morbidity due to malaria in Cameroon was 40.1% among children under 5 and 22.5% for pregnant women [1]. Malaria transmission is highly heterogeneous in the country, with high and perennial parasite transmission occurring in the forest, coastal and humid savanna areas; low parasite transmission in the highlands; and seasonal parasite transmission in the sahelian and dry savanna areas [1]. *Plasmodium falciparum* is the main parasite responsible for more than 95% of the cases [2] followed by other human-infecting *Plasmodium* species including *P. malariae*, *P. ovale* and *P. vivax* [3]. Up to 52 *Anopheles* species have been recorded across the eco-epidemiological zones of the country [4]. Around 16 species have been demonstrated to support the development and spread the human *Plasmodium* spp., among which 6 main vectors, namely *An. gambiae* (s.s.), *An. coluzzii*, *An. arabiensis*, *An. funestus*, *An. nili* and *An. moucheti* [3, 5].

In Cameroon, over the last decade, significant reduction in malaria burden has been recorded. Morbidity and mortality decreased from 36 to 28% and from 31 to 18.3% respectively [1], mainly due to the scale-up of long-lasting insecticide nets (LLINs), which significantly reduce the number of female malaria vectors population that takes blood meals and subsequently rest indoor [1, 6]. Unfortunately, from 2015 to date, a significant increase in malaria incidence has been observed due to widespread physiological resistance and behavioural changes, with the country still being one of the 11 high worldwide burden countries [7–9]. In 2017, all 10 high burden African countries reported an increase in malaria cases compared to the previous year, ranging from an estimated 131 000 more cases in Cameroon to 1.3 million additional cases in Nigeria [9].

Insecticide resistance (IR) is undermining current vector control interventions [10]. In insect populations, IR is predominantly based on enzymatic detoxification as well as by the alteration of insecticide target sites leading to insecticide insensitivity [6, 11]. Metabolic mechanisms are the result of over-expression, either by amplification and/or up-regulation of detoxification genes (Cytochrome P450s, glutathione S-transferases and esterases) [12]. This type of resistance has been consistently reported to be the main driver of pyrethroids and DDT resistance in the malaria vector *An. funestus* s.l, since no kdr mutation has been detected so far in this species [13, 14]. Despite increasing reports of pyrethroid resistance in malaria vectors, little is known about the impact of metabolic resistance mechanisms on malaria transmission. The lack of resistance markers, particularly for metabolic resistance, has been one of the limiting factors preventing a robust assessment of the resistance impact, notably through application of entomological parameters. The detection of a single amino acid change (L119F) in the glutathione S-transferase epsilon 2 (*GSTe2*) gene conferring DDT/pyrethroid resistance in *An. funestus* [15] helped to start assessing the impact of metabolic resistance on *An. funestus* infectivity [14]. The study revealed that field-collected mosquitoes homozygous resistant for the L119F mutation were more likely to harbour the *Plasmodium* parasite and develop it to the infective stage, suggesting that, this gene could exacerbate malaria transmission in the field, with important public health consequences. However, it is still unclear how this marker could affect the mosquito vectorial capacity, including other important malaria transmission indices such as mosquito biting rate, parity and the entomological inoculation rate (EIR).

Mosquitoes have also been shown to adapt to a changing environment through behavioural avoidance undermining vector control tools efforts and resulting in the increase of residual transmission [7]. Behavioural modifications include changes in biting time and place, changes in host choice and a shift from endophilic to exophilic behaviour [8]. Vector behavioural changes and resistance selection are important parameters that could play a greater role in the persistent malaria transmission and can only be captured by real-time surveillance [16]. It is now recommended that the deployment of vector control tools is followed by rigorous resistance monitoring and routine entomological and epidemiological surveillance activities [4, 7]. Undertaking such research studies could help to better understand the insecticide resistance status; ecology, behaviour and dynamics of local vector populations; mosquito biting rates; entomological inoculation rates (EIR) and changes in malaria incidence, which could explain residual/persistent malaria transmission and provide avenues for the development of appropriate resistance management strategies. The present study sought to assess how L119F-*Gste2* Conferring metabolic resistance influences entomological parameters underpinning *An. funestus*’ propensity to transmit *Plasmodium* spp.

## Methods

### Study site

**Fig. 1:**
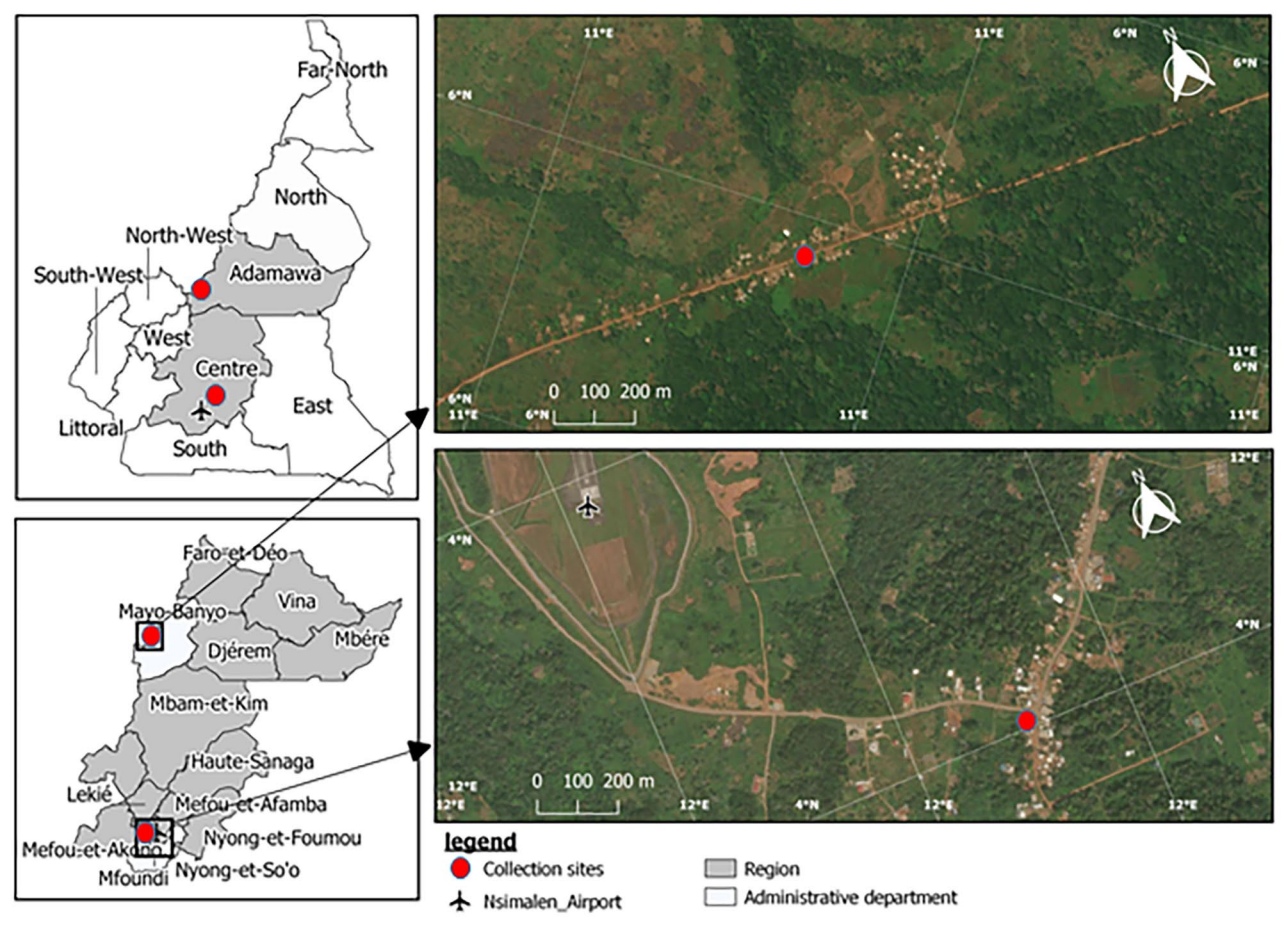
Map of the study sites

The study was conducted in two different eco-epidemiological settings in Cameroon: Elende (3°41’57.27’’N, 11°33’28.46’’E) in the humid equatorial forest domain and Mibellon (6^o^ 460 N, 11^o^ 700 E) in the humid savannah (Fig. 1). Both villages are located in the Centre Region, Nyong-Et-Soo Division, Nkolmefou I SubDivision; and in the Adamawa Region, Mayo Banyo Division, Bankim Subdivision, respectively.

Elende is a rural village located about 50 km South-East of Yaoundé (the country’s capital city) and about 2 km from the Yaoundé-Nsimalen International Airport. Located close to the Mefou River, this village is characterised by a classic Guinean equatorial climate with four distinct seasons: a short rainy season from mid-March to the end of June; a short dry season from late June to mid-August; a long rainy season which runs from mid-August to mid-November and a long dry season which runs from mid-November to mid-March [17]. The average temperature is about 21 °C. Most of the agricultural assets are used for subsistence farming, with the main crops being cassava, cocoa, plantain, maize and vegetables. The yield of these agricultural practices is largely enhanced by the intensive use of pesticides [17]. Most houses are made mainly of brick followed by mud walls, with iron sheets and sometimes thatched roofs. Some parameters, such as the strong human influence on the vegetation and the closeness of the village to marshy lands and streams joining major rivers, create temporary and permanent breeding sites for malaria vectors such as *An. funestus* s.l and *Anopheles gambiae* s.l which represent the predominant malaria vectors. Pyrethroids- containing LLINs are the main prevention method with a coverage of around 60% [17]. Before the scale up of LLINs in the Region, EIR was known to vary between 100 and 350 infective bites/person/year [4].

Mibellon is a rural village located 350 km from Yaoundé the capital city of Cameroon. The climate is Sudano-Guinean, characterised by a five-month rainy season from March to April and from August to October, and a seven-month dry season extending from November to February and from May to July [18]. The average temperature is about 23 °C. Human activities are mainly fishing, hunting and subsistence farming, including maize, watermelon and coffee plantations. Most houses are made mainly of mud and brick walls, with thatched or iron sheet roofs [19]. Mibellon is close to permanent water bodies, including a lake and swamps, which provide suitable breeding sites for mosquitoe. Two main malaria vector species namely *An. gambia*e s.l and *An. funestus* s.l are routinely found in the village, with the latter being the most abundant throughout the year and playing a greater role in malaria transmission [18]. The main vector control approach is LLINs, and the villages benefited from the universal LLIN coverage campaigns in the recent years (2015) before this study. A survey in the area revealed a high use of insecticides in the coffee and watermelon farms [18]. These insecticides are mainly pyrethroids, neonicotinoids and carbamates. The main malaria vectors found there are resistant to pyrethroids and DDT [18]. The region could be classified as belonging to a mesoendemic stratum with perennial malaria parasite transmission.

### Household choice

The inhabited households where mosquito collection was undertaken were randomly selected after obtaining the consent from the head of the household. They were assembled according to trapping methods [Human Landing Catches (HLCs, 5 houses), CDC Light Traps (CDC-LTs, 5 houses), Pyrethrum Spray Catches (PSCs, 20 houses)], and a distance of 50–200 m was maintained between each collection point. These houses were made of mud or cement with traditional roof or sheet metal and were representative of the local habitat.

### *Anopheles* species sampling

Wild mosquitoes were caught every month during one year in Elende [from December 2019 to November 2020 except 3 months (from April to June) due to the Covid-19 pandemic]; and bi-monthly the following year in Mibellon (January, March, May, July, September and November 2021) using three collection methods including HLCs, CDC-LTs and PSCs.

#### Human landing catches

Monthly HLCs were performed inside and outside five houses during three consecutive nights to collect adult mosquitoes landing on human baits. Collections were carried-out between 6:00 pm and 6:00 am to study the change in vector behaviour. A team of four trained volunteers per house (two working during the first half of the night and the others during the second half of the night) carried out the collection, one sitting inside the house and the other on the veranda, with their legs exposed to attract host-seeking mosquitoes. A flashlight was used to locate and dazzle mosquitoes landing on the exposed lower limbs of the collectors, while haemolysis tubes were used to collect mosquitoes before they could bite. The tubes were covered with cotton after individual collection and transferred every hour to custom-made bags for a total of 12 h. Volunteers were offered treatment if they developed symptoms of malaria during the study.

#### CDC light traps

Ten light traps were installed each month during three consecutive nights, one indoors and another outdoors of five houses in each site at each collection period between 6:00 p.m. and 6:00 a.m. In each bedroom, the trap was hung 1.5 m above the floor near a bed (on the foot side) covered with a mosquito net and where at least one household member was spending the night. Another trap was hung in the same way outside near the window of the same house. Two supervisors were recruited to ensure that the traps set remained operational during all the collection period. Collection boxes were retrieved from traps in each house in the morning from 7:00 am to 8:00 am by the collection team.

#### Pyrethrum spray catches

The PSCs were carried out each month during morning hours, between 6:00 a.m. and 10:00 a.m. to collect indoor resting mosquitoes for two consecutive days in 10 sleeping rooms. Walls and roofs were sprayed with the commercial pyrethroid + piperonyl butoxide (PBO) insecticide. Houses with open eaves were sprayed from outside through the eaves before entering and spraying indoors. After 10 min, all female anopheline species knocked down by the chemical were collected from the white sheets, transferred inside petri dishes and then sorted according to the physiological state of their abdomen (unfed, blood-fed, half-gravid and gravid). To determine blood meal source, blood-fed female anophelines mosquitoes were kept individually in labelled Eppendorf tubes for further laboratory analysis.

### Ovary dissection

Ovaries of unfed *Anopheles* mosquitoes (about 20% of the total) collected at hourly night intervals with HLC technique were dissected directly after collection on the field, on a sterile microscope slide in distilled water. Binocular dissection microscope at 10X magnification were used to look for the presence of tightly coiled skeins or loose coils, indicative of a nulliparous or parous ovary, respectively.

### Mosquito species identification

Collected mosquitoes were sorted into anophelines and culicines genus. All *Anopheles* mosquitoes were identified morphologically on the field using appropriate identification keys [19]. Once in the laboratory, genomic DNA was extracted from head/thorax and abdomen separately of all collected mosquitoes following the Livak method [20]. The DNA extracted from the head/thorax was quantified using NanoDrop™ spectrophotometer (Thermo Scientifc, Wilmington, USA) and species-specific polymerase chain reaction (PCR) based on the protocols of Koekemoer et al. [21], Santomalazza et al. [22] and Kengne et al. [23] were used to identify sibling species of *An. funestus* group, *An. gambiae* complex and *An. nili* goup respectively.

### Estimation of *Plasmodium* infection rate

The gDNA extracted from the head and thoraces of *Anopheles* vectors collected indoor and outdoor through HLC method were analysed by the TaqMan assay described by Bass et al. [24], using MX 3005 (Agilent, Santa Clara, CA, USA) to detect the presence of *Plasmodium* parasites. All the positive samples by TaqMan were used to confirm and discriminate *Plasmodium* species according to the Nested PCR based of Snounou et al. [25], with slight modifications consisting in using kappa Taq enzyme instead of Ampli Taq [26] and a multiplex detection of *Plasmodium* species by mixing specific-species primers during the second cycle of amplification.

### Genotyping of L119F-*GSTe2* mutation in *An. funestus* s.s

The allele-specific PCR (AS-PCR) method was used for the detection of the L119F-*GSTe2* marker (associated with cross-resistance to DDT and pyrethroids) in field collected *An. funestus* s.s [15, 27]from HLC technic only. This choice was to clearly establish the impact of the marker on all the transmission parameters because mosquitoes collected in the CDC-LT in the morning were usually dead and dissection was not possible. The amplified products corresponding to different genotypes were separated by agarose gel electrophoresis (2%) following the procedure described by Tchouakui et al. [27]. In addition, the potential association between this marker and malaria transmission entomological parameters was assessed using odds ratio and Fisher exact test.

### Detection of blood meal source

The engorged abdomens of field mosquitoes collected using PSCs method were used for ELISA blood meal origins. Monoclonal antibodies against human, cow, cattle, pig, horse, chicken, sheep, dog and rat blood were used following the procedure described by Beier et al. [28]

### Data analysis

The GraphPad Prism (version 8.0.2) and Stata 16.1 software were used to analyse the data. Pearson’s Chi square tests and Incidence Rate Ratio were used to compare proportions or rates. Approximately 50% of the *An. funestus* s.s collected from the HLC technique were used to determine the association between the *GSTe2* mutation and malaria transmission parameters during each collection month. The other field-collected anopheline species were all analysed only for their entomological parameters (the human biting rate, human blood index, sporozoite infection rate and the entomological inoculation rate), which were estimated using formulas described in the Additional file 1 (Table S1). Association between the *GSTe2* mutation and malaria transmission parameters was assessed by comparing the Odds Ratio/Incidence Rate Ratio of malaria transmission parameter between the homozygous resistant (119 F/F), heterozygote (L119F-RS) and homozygous susceptible (L/L119) individuals.

## Results

### *Anopheles* mosquito species composition and abundance

**Fig. 2:**
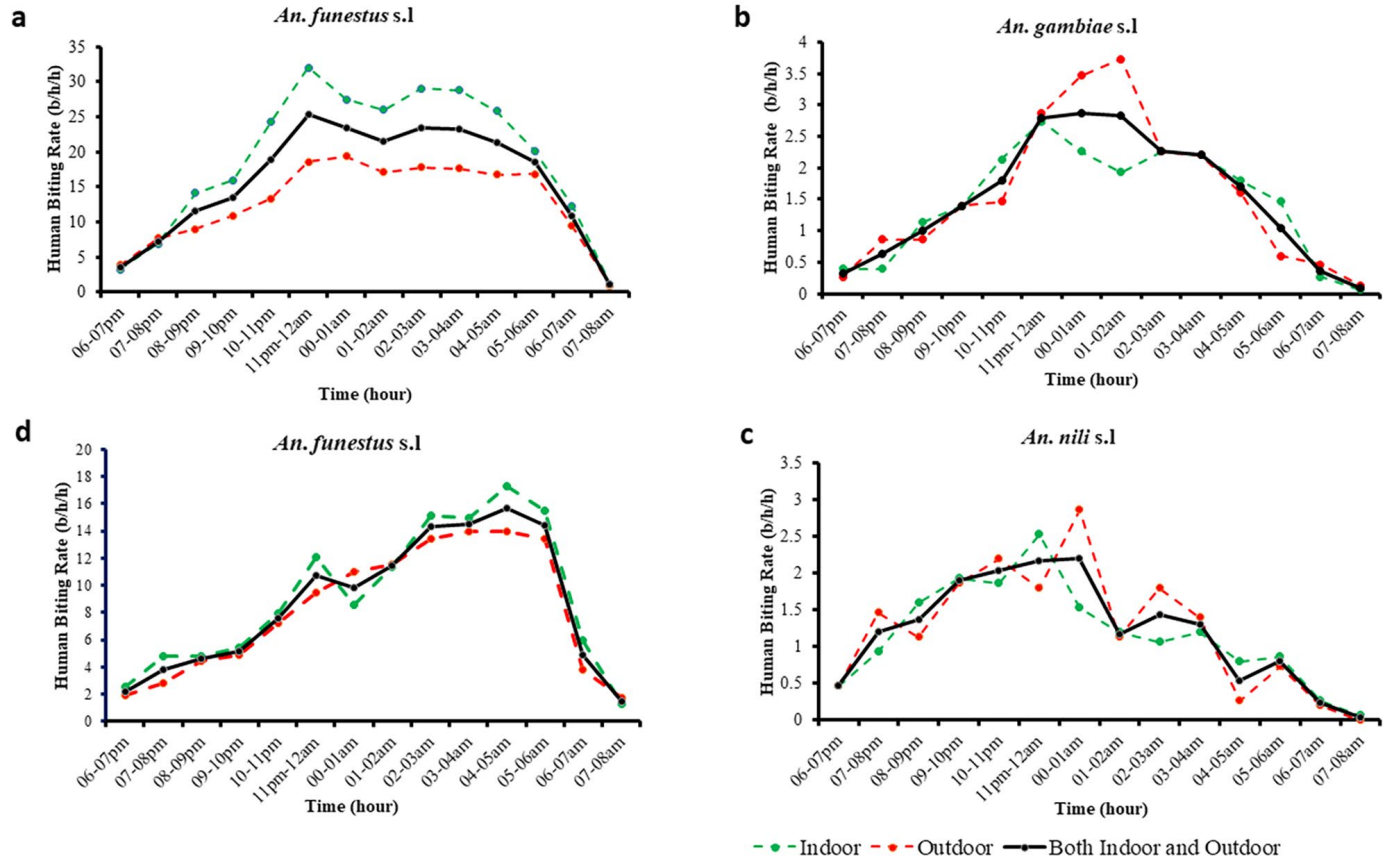
Anopheline mosquito species composition after longitudinal survey in Elende and Mibellon from 2019–2021. **a & e**: Global species composition. **b & f**: species composition within the *Anopheles funestus* group. **c & g**: species composition within the *Anopheles gambiae* complex. **d**: species composition within the *Anopheles nili* group

A total of 15,946 mosquitoes belonging to the *Anopheles* genus were sampled in both sites using HLC, CDC_LT and PSC methods. HLC was by far the best collection method regardless the collection site, with 82 and 57% of the total number of *Anopheles* mosquitoes collected in Elende and Mibellon, respectively. The Anopheline fauna collected in Elende was dominated by *An. funestus* s.l (8088/9341 [86.6%]) followed by *An. gambiae* s.l (701/9341 [7.5%]). This was the same in Mibellon (6364/6604 [96.4%]; 158/6604 [2.4%] for the same species, respectively) (Table 1). Others species included *An. nili* (527/9341 [5.6%] and *An. ziemanni* (25/9341 [0.3%] in Elende; then *An. rufipes* (50/6604 [0.7%]) and *An. ziemanni* (32/6604 [0.5%]) in Mibellon (Table 1). In Elende, molecular identification using PCR revealed the presence of *An. funestus* s.s (2050/2074 [99%]) and *An. leesoni* (24/2074 [1%]) as members of the *An. funestus* group; then *An. gambiae s.s* (344/403 [85%]) and *An. coluzzii* (59/403 [15%]) as members of the *An. gambiae* complex; and *An. nili* (313/313 [100%]) as the sole member of the *An. nili* group. In Mibellon, *An. funestus* s.s (344/347 [99%]), *An. leesoni* (2/347 [0.6%]) and *An. rivulorum* (1/347 [0.4%]) were members of the *An. funestus* group; whereas *An. gambiae s.s* (89/93 [96%]) and *An. arabiensis* (4/93 [4%]) were identified as members of the *An. gambiae* complex (Fig. 2).

**Table 1:** Anopheline species composition in Elende and Mibellon

Sites	Species	Collection technics							
		HLC		CDC-LT		PSC		Total	
		n	%	n	%	n	%	n	%
Elende	*An. funestus* s.l	6703	85.2	415	90	970	96.2	8088	86.6
	*An. gambiae* s.l	640	8.1	25	5.4	36	3.6	701	7.5
	*An. nili* s.l	504	6.4	21	4.5	2	0.2	527	5.6
	*An. ziemanni*	24	0.3	1	0.2	0	0	25	0.3
	Total	7871	100	462	100	1008	100	9341	100
Mibellon	*An. funestus* s.l	3642	96.5	1448	95.6	1274	96.7	6364	96.4
	*An. gambiae* s.l	105	2.8	20	1.3	33	2.5	158	2.4
	A*n. rufipes*	18	0.5	24	1.6	8	0.6	50	0.7
	*An. ziemanni*	8	0.2	22	1.5	2	0.2	32	0.5
	Total	3773	100	1514	100	1317	100	6604	100

n = Mosquito abundance

### Parity rate

**Fig. 3:**
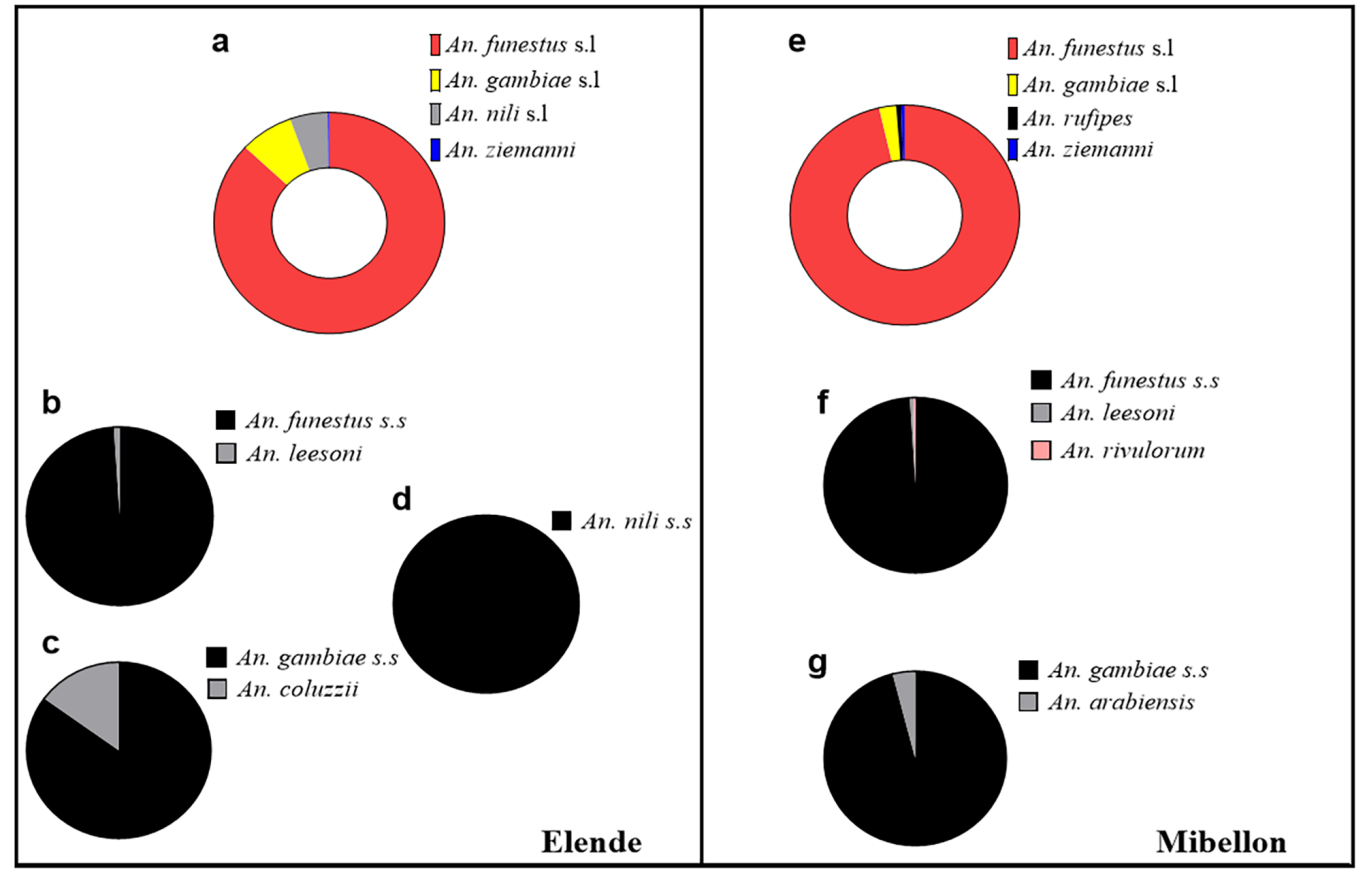
Parity rate of *Anopheles* mosquitoes collected per month from 2019–2021. **a**: Elende, **b**: Mibellon

Regardless of collection site and months, the parity rate of all anopheline mosquitoes collected was very high, ranging from 52 to 100% in Elende **(**Fig. 3a**)** and from 58 to 75% in Mibellon (Fig. 3b), with an overall parity rate of 66.1%. *An. funestus* s.l from Mibellon was significantly more parous than the same species (IRR (Incidence Rate Ratio) = 1.2, 95%CI (1.02–1.3), p = 0.02) or all species combined in Elende (IRR = 1.1, 95%CI (1.01–1.3), p = 0.04) (Additional file 1, Table S2).

### Human blood source

**Fig. 4:**
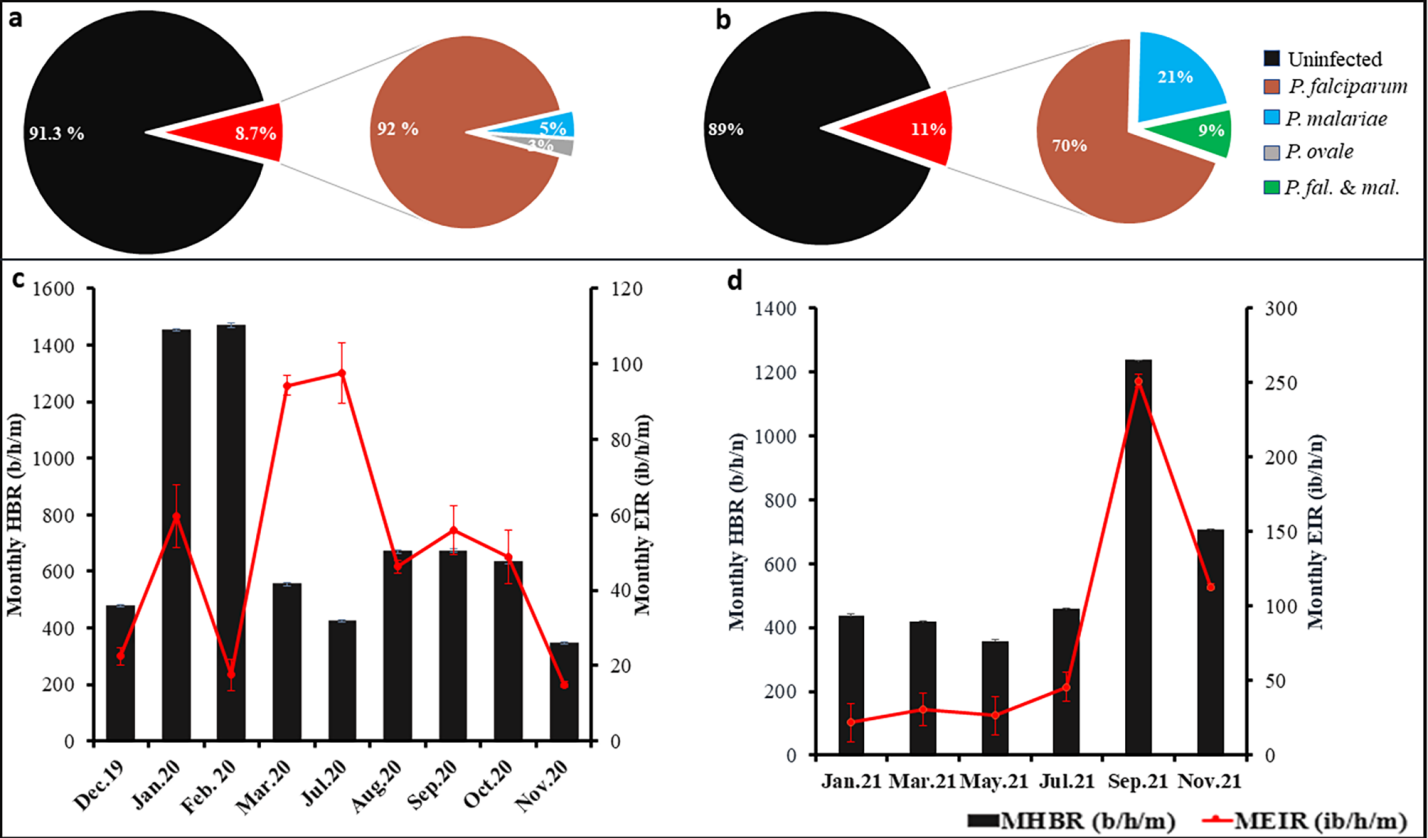
Feeding behaviour of Anopheline species in Elende and Mibellon after longitudinal survey from 2019–2021. **a**: Proportion of host preference by *Anopheles* species in Elende. **b**: Proportion of host preference by *Anopheles* species in Mibellon

The 87.8% (252/287) of mosquitoes tested for blood meal origin were positive for the presence of vertebrate’s blood antigens. In Mibellon, the most frequent blood meal was human (60.2%) followed by dog (33.8%), pig (3.5%) and sheep (2.5%) (Fig. 4b). Mixed blood from 2, 3 and 4 different vertebrate’s host were detected in *An. funestus* s.l and *An. gambiae* s.l (Additional file 1, Table S3). In Elende, mosquitoes showed preference only for two hosts, human (94%) the predominant one, and sheep (6%) (Fig. 4a). Unlike to Mibellon, no multiple blood meal source was detected in *An. gambiae* s.l and *An. funestus* s.l. All the *Anopheles* mosquitoes analysed fed mainly on human with a very high human blood index (HBI = 94%) in each site (Table 2). In Mibellon, the highest HBI was found in *An. rufipes* (100%) followed by *An. funestus* s.l (97.6%) and *An. gambiae* s.l (50%). Beside this, a relatively high feeding rate on dog (53%) was also detected (Table 2). In Elende, the highest HBI was found in *An. funestus* s.l (98%) followed by *An. gambiae* s.l (75%) (Table 2). No significant difference was observed between HBIs of *An. gambiae* s.l (IRR = 1.5, 95%CI (0.4–4.7), p = 0.4) and *An. funestus* s.l (IRR = 1.002, 95%CI (0.7–1.4), p = 0.9) across the two collection sites.

**Table 2:** Blood meal origin of *Anopheles* vectors collected by PSC in Elende and Mibellon

Sites	Species	Total tested (Positive and Negative for blood Elisa)	Tested Positive for Blood antigene of specific vertebrate host (single and Mixed)				Tested Unknown	HBI (%)	Feeding rate on Dog (%)
Mibellon			Human (%)	Dog (%)	Pig (%)	Sheep (%)			
	*An. funestus* s.l	212	207 (97.6)	113 (53.3)	11 (5.2)	8(3.8)	5	97.6	53.3
	*An. gambiae* s.l	18	9 (50)	10 (55.6)	2 (11.1)	1 (5.6)	0	50	55.5
	*An. rufipes*	3	3 (100)	0 (0)	0 (0)	0 (0)	0	100	0
	Total tested	233	219 (94)	123 (53)	13 (5.6)	9 (4)	5	94	53
Elende	*An. funestus* s.l	46	45 (98)	NA	NA	1 (2.2)	0	98	NA
	*An. gambiae* s.l	8	6 (75)	NA	NA	2 (25)	0	75	NA
	Total tested	54	51 (94)	NA	NA	3 (6)	0	94	NA

HBI = Human Blood Index, NA = Not Applicable

### Hourly biting pattern and monthly human biting rates (MHBRs) of anopheline mosquitoes

**Fig. 5:**
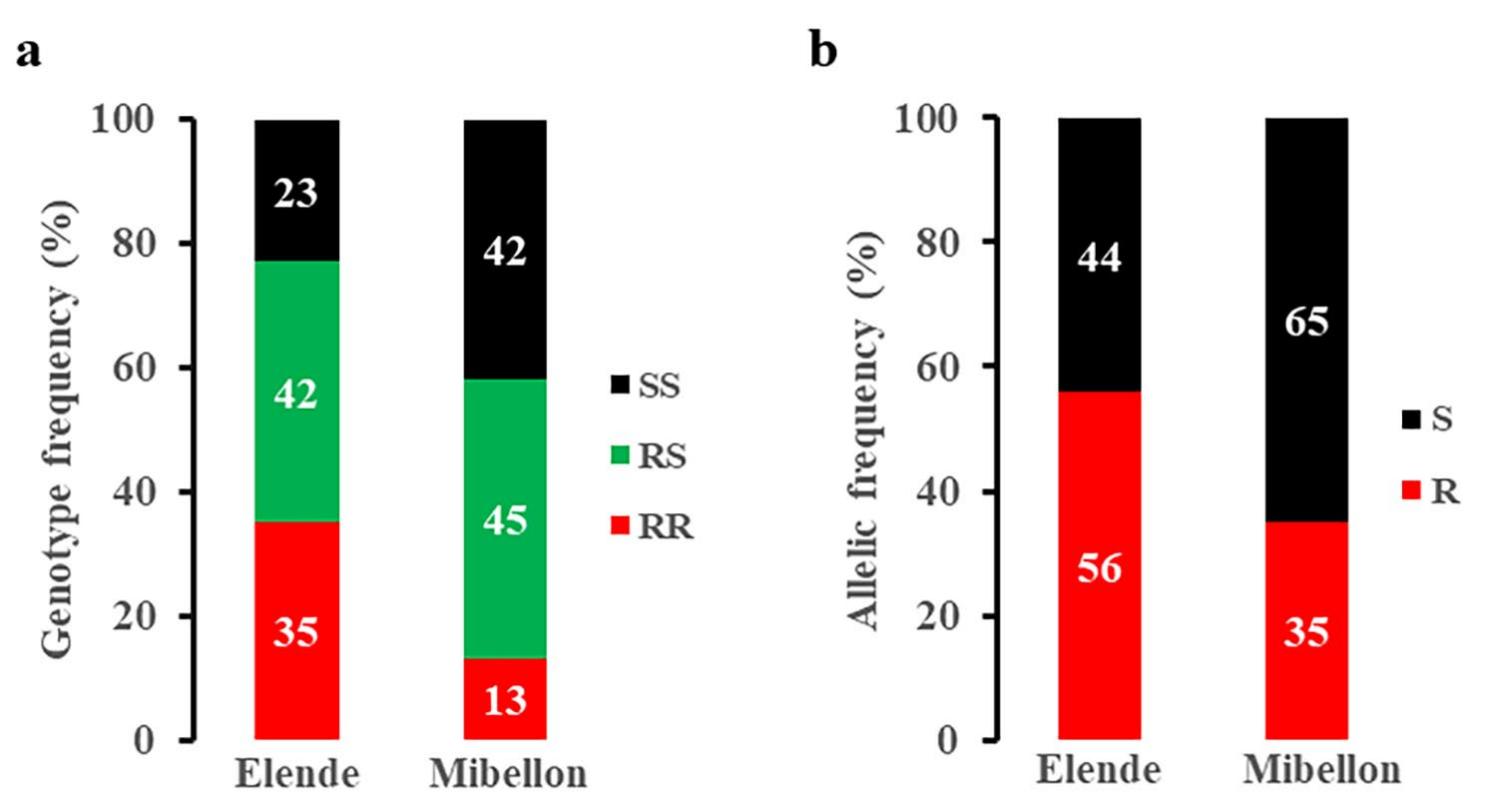
Hourly biting activities of Anopheline mosquitoes. **a, b & c**: Elende; **d**: Mibellon

All *Anopheles* species collected were aggressive throughout the night both indoors and outdoors. *Anopheles* biting activity started early in the evening (6 p.m.) and ended very late in the morning (7 a.m.), with some bites recorded until 8 a.m. for *An. funestus* s.l. irrespective of the collection site (Fig. 5a & d). In Elende, the peak biting activity of *An. funestus* s.l was recorded during the first part of the night (between 11p.m.-00a.m.), while that of *An. gambiae* s.l and *An. nili* s.l was recorded during the second part of the night (both between 00-01a.m.) (Fig. 5b & c). In contrast to Elende, the peak biting activity of *An. funestus* s.l from Mibellon was recorded during the second part of the night (between 04-05a.m.). Overall, the hourly biting activity of *An. funestus* s.l., *An. gambiae* s.l. from Elende; and *An. funestus* s.l. from Mibellon was significantly higher (IRR = 2, 95%CI [1.7–2.8], p < 0.0001; IRR = 1. 7, 95%CI [1.4-2], p < 0.0001 and IRR = 2.5, 95%CI [2.3–2.7], p < 0.0001 respectively) in the second part of the night (from 00a.m. to 08a.m.) compared to the first part (from 6p.m. to 00a.m.).

**Fig. 6:**
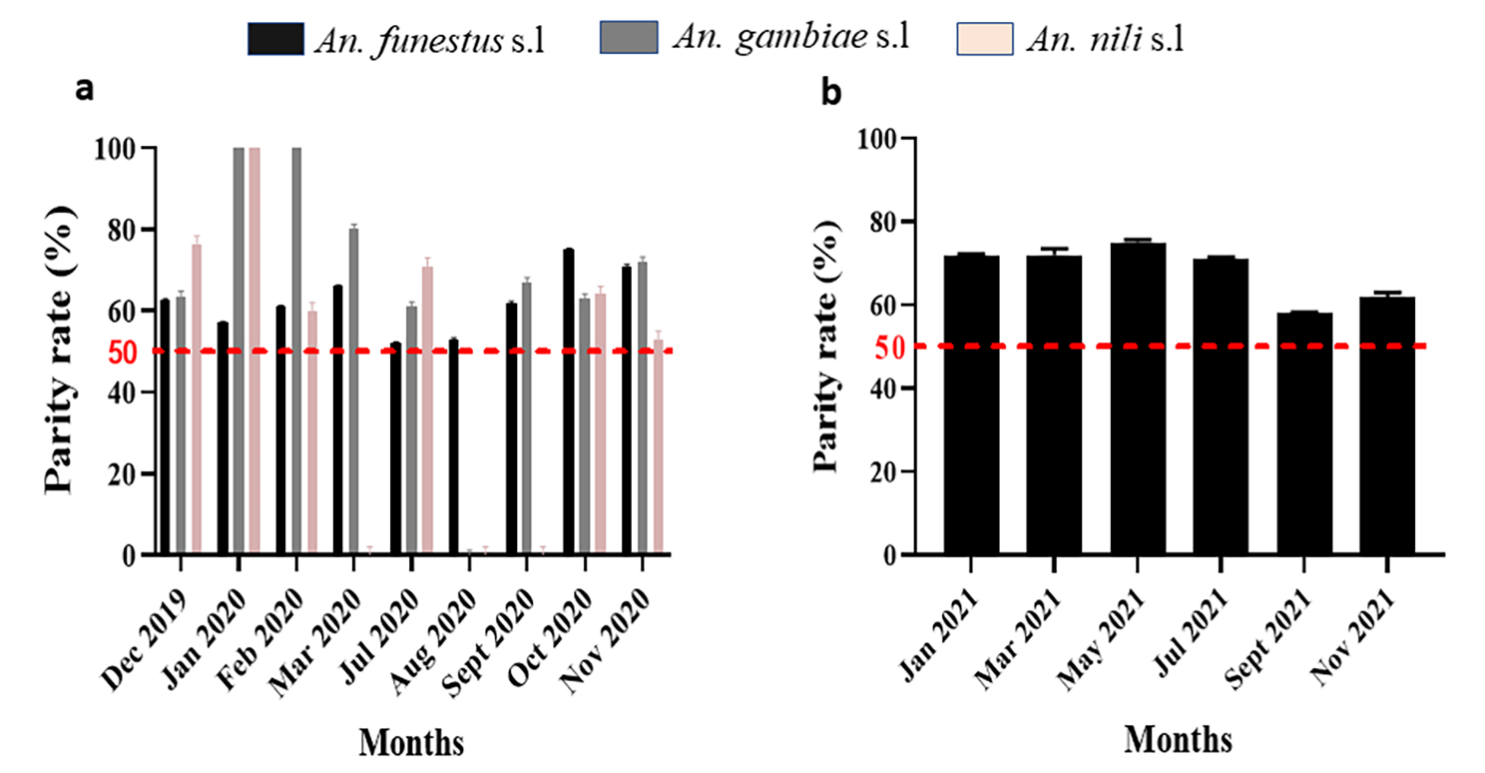
Main malariometric indices associated with *An. funestus* s.l. in Elende and Mibellon from 2019–2021. **a & c**: Sporozoite infection rate, Monthly human biting rate, and Entomological inoculation rate in Elende; **b & d**: Sporozoite infection rate, Monthly human biting rate and Entomological inoculation rate in Mibellon

In Elende, the highest MHBRs of *An. funestus* s.l. were observed in January and February 2020 (Fig. 6c), and the mean MHBR was significantly higher indoors (891 b/h/m) compared to outdoors (600 b/h/m) (IRR = 1.5, 95%CI [1.4–1.6], p < 0.001). However, there was no significant difference between the mean indoor (60 b/h/m) and outdoor (63 b/h/m) MHBR of *An. gambiae* s.l. at the same site (IRR = 1.1, 95%CI [0.9–1.3], p = 0.5). In Mibellon, the highest MHBRs of *An. funestus* s.l. were observed in September and November 2021 (Fig. 6d). As observed in Elende, the mean MHBRs of *An. funestus* s.l. from Mibellon were significantly higher indoors (639 b/h/m) compared to outdoors (570 b/h/m) (IRR = 1.2, 95%CI [1.1-3. 6], p = 0.005) and there was no significant difference between the mean indoor (21 b/h/m) and outdoor (12 b/h/m) MHBR of *An. gambiae* s.l. at the same site (IRR = 1.2, 95%CI [0.8–1.8], p = 0.3). Globally, the MHBR of *An. funestus* s.l. was significantly higher in Elende (750 b/h/m) than in Mibellon (606 b/h/m) (IRR = 1.2, 95%CI [1.25–1.3], p < 0.001). The same result was observed for *An. gambiae* s.l. (36 b/h/m and 18 b/h/m in the same site, respectively) (IRR = 3.6, 95%CI [3-4.5], p < 0.001) (Table 3).

**Table 3:** Entomological indices of the main malaria vectors collected by HLC in Elende.and Mibellon

Species	Sites	Biting Place	SIR (%) 95%CI (Tested)	IRR (95%CI)	p-value	MHBR (b/h/m)	IRR (95%CI)	p-value	MEIR (ib/h/m)	IRR (95%CI)	p-value
*An. funestus* s.l	Elende	Indoor	10.3 [7–14] (446)	1.6 [0.9-3]	0.07	891	1.5 [1.4–1.6]	< 0.001^**^	93	2.3 [1.9–2.9]	< 0.001^**^
		Outdoor	6.5 [4–10] (309)			600			39		
		**Total**	**8.7 [6.7–11] (755)**			**750**			**66**		
	Mibellon	Indoor	12.8 [10.5–15.5] (838)	1.5 [1.1–2.1]	0.01^*^	639	1.2 [1.1–3.6]	< 0.005^**^	81	1.7 [1.4–2.1]	< 0.001^**^
		Outdoor	8.4 [6.4–10.7] (739)			570			48		
		**Total**	**11 [9.2–12.5] (1577)**			**606**			**66**		
*An. gambiae* s.l	Elende	Indoor	3.6 [0.9–9] (110)	2.9 [0.3–146]	0.3	60	1.1 [0.9–1.3]	0.5	2.1	2.3 [0.5–14]	0.2
		Outdoor	1.2 [0.03–6] (82)			63			1		
		**Total**	**2.6 [0.8–6] (192)**			**60**			**1.5**		
	Mibellon	Indoor	3.3 [0.4–11] (61)	NA	NA	21	1.2 [0.8–1.8]	0.3	0.6	NA	NA
		Outdoor	0 [0] (30)			12			0		
		**Total**	**2.2 [0.2–7] (91)**			**18**			**0.3**		

b/h/n: bites/human/night; b/h/m: bites/human/month; ib/h/n: infected bite/human/night; ib/h/m: infected bite/human/month

### Sporozoite infection rates (SIRs) and entomological inoculation rates (EIRs) of *Anopheles funestus* s.l. and *Anopheles gambiae* s.l. mosquitoes collected by human-landing catch

The overall infection rates of *An. funestus* s.l. mosquitoes by sporozoites of different plasmodial species were very high in both collection sites (8.7 and 11% in Elende and Mibellon, respectively) (Fig. 6a&b), whereas those of *An. gambiae* s.l. were much lower (2.6 and 2.2% in the same sites, respectively) (Table 3). The comparison between indoor and outdoor SIRs showed that there was no significant difference for *An. funestus* s.l. (IRR = 1.6, 95%CI [0.9-3], p = 0.07) and *An. gambiae* s.l. (IRR = 2.9, 95%CI [0.3–146], p = 0.3) from Elende. However, *An. funestus* s.l. from Mibellon was significantly more infected indoors than outdoors (IRR = 1.5, 95%CI [1.1–2.1], p = 0.01) (Table 3). The SIRs of *An. funestus* s.l. varied too much according to the months of the survey. In Elende, the SIRs ranged from 1.2 to 23%, with the highest rate detected in July (23%) and March 2020 (17%), whereas in Mibellon, the SIRs varied from 5 to 20%, with the highest rate detected in September (20%) and November 2021 (16%) (Additional file 1, Table S4). However, despite these variations by survey month, the SIR of *An. funestus* s.l. was not significantly different between the wet and dry seasons (IRR = 1.4, 95%CI [0.8–2.3], p = 0.2; IRR = 1.02, 95%CI [0.7–1.4], p = 0.9 in Elende and Mibellon, respectively). *Plasmodium falciparum* was by far the predominant species infecting *An. funestus* s.l. in both sites, accounting for 92 and 70% of total infections in Elende and Mibellon respectively, while *P. malariae* accounted for 5 and 21% in the same sites. Other species, including *P. ovale* infection (3%) and *P. falciparum/P. malariae* co-infection (9%), were detected only in Elende and Mibellon (Fig. 6a&b). Despite differences in the *Plasmodium* species infecting *An. funestus* s.l. between sites, there was no significant difference in the overall SIR of *An. funestus* s.l. between the two study sites (IRR = 1.2, 95%CI [0.9–1.6], p = 0.1). *P. falciparum* was the only species infecting the salivary gland of *An. gambiae* s.l. in both sites, in contrast to *An. funestus* s.l. Globally, in Elende, *An. funestus* s.l. supported 93% (66/71) of all sporozoite infection events, while *An. gambiae* s.l. supported only 7% (5/71). However, in Mibellon, 98.8% of sporozoite infection events were supported by *An. funestus* s.l. while only 1.2% were supported by *An. gambiae* s.l. Regardless of the site, 97% (236/243) of all sporozoite infection events at both sites were supported by *An. funestus* s.l. while only 3% were supported by *An. gambiae* s.l. No infection was detected in the other *Anopheles* species collected during the study period.

The overall monthly EIR of *An. funestus* s.l. was very high and exactly equal at both sites (66 ib/h/m), while *An. gambiae* s.l. was associated with very low monthly malaria transmission intensities at both sites (1.5 and 0.3 ib/h/m in Elende and Mibellon, respectively). Regardless of the site, indoor monthly EIRs (93 and 81 ib/h/m) associated with *An. funestus* s.l. were significantly higher than outdoor monthly EIR (39 and 41 ib/h/m) (IRR = 2.3, 95%CI [1.9–2.9], p < 0.001; IRR = 1.7, 95%CI [1.4–2.1], p < 0.001 in Elende and Mibellon, respectively) (Table 3). This was not applied to *An. gambiae* s.l. as no significant difference were observed between indoor and outdoor transmission intensities (Table 3). As with HBRs, EIRs of *An. funestus* s.l. were also subject to monthly variations, with the highest EIRs observed in the same months as the monthly HBRs in Mibellon, but in different months in Elende, notably July (98 ib/h/m) and March 2020 (94 ib/h/m) (Fig. 6c&d). Despite this latter observation, there was no significant difference between the malaria transmission intensities of *An. funestus* s.l. recorded during the wet and dry seasons, irrespective of the site (IRR = 1.2, 95%CI [0.9–1.4], p = 0.09; IRR = 1.1, 95%CI [0.9–1.3], p = 0.3 in Elende and Mibellon, respectively). Overall, *An. funestus* s.l. was responsible for 98.6% of all malaria transmission events at both sites, while *An. gambiae* s.l. played a minor role (1.4%).

### Distribution of the L119F-*GSTe2* gene in *An. funestus* s.s mosquitoes and its influence on malaria transmission in Elende and Mibellon

**Fig. 7:**
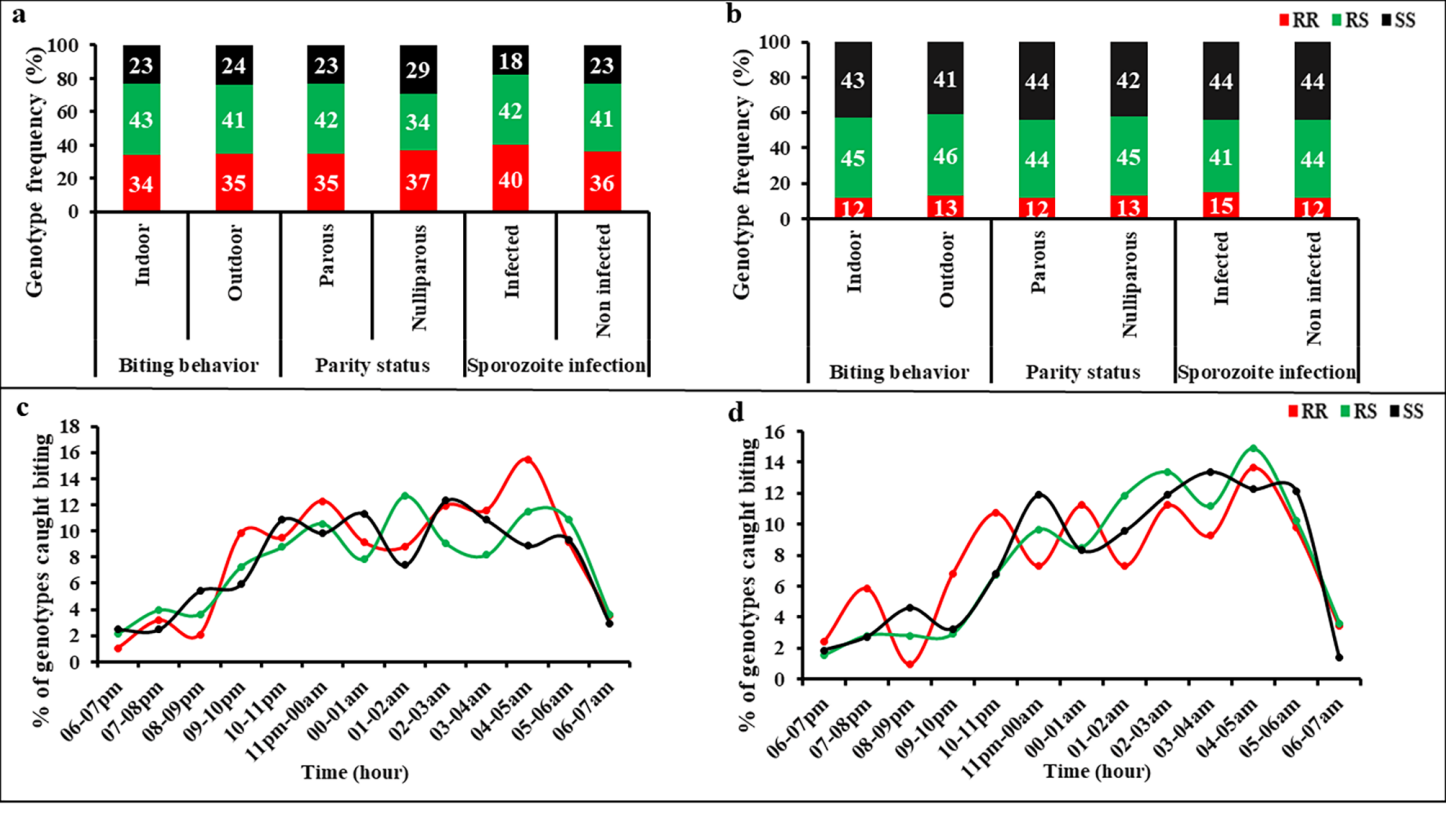
Global distribution of L119F-*GSTe2* metabolic resistance marker in Elende and Mibellon. **a**: Genotypes distribution; **b**: Allelic distribution. **RR**: 119 F/F homozygous resistant; **RS**: L119F heterozygous; **SS**: L/L119 homozygous susceptible; **R**: 119 F resistant allele; **S**: L119 susceptible allele

Following monthly collections, a total of 3630 mosquitoes (1744 and 1886 from Elende and Mibellon respectively) were genotyped for the L119F-*Gste2* gene and the annual distribution of each genotype and allele is presented in Fig. 7. Data revealed a significant lower frequency of the 119 F resistant allele in the *An. funestus* s.s. population from Mibellon (35%) compared to Elende (56%) (IRR = 1.8, 95%IC [1.7-2], p < 0.001).

#### Association between the L119F-*GSTe2* resistant marker and *An. funestus’* parity

**Fig. 8:**
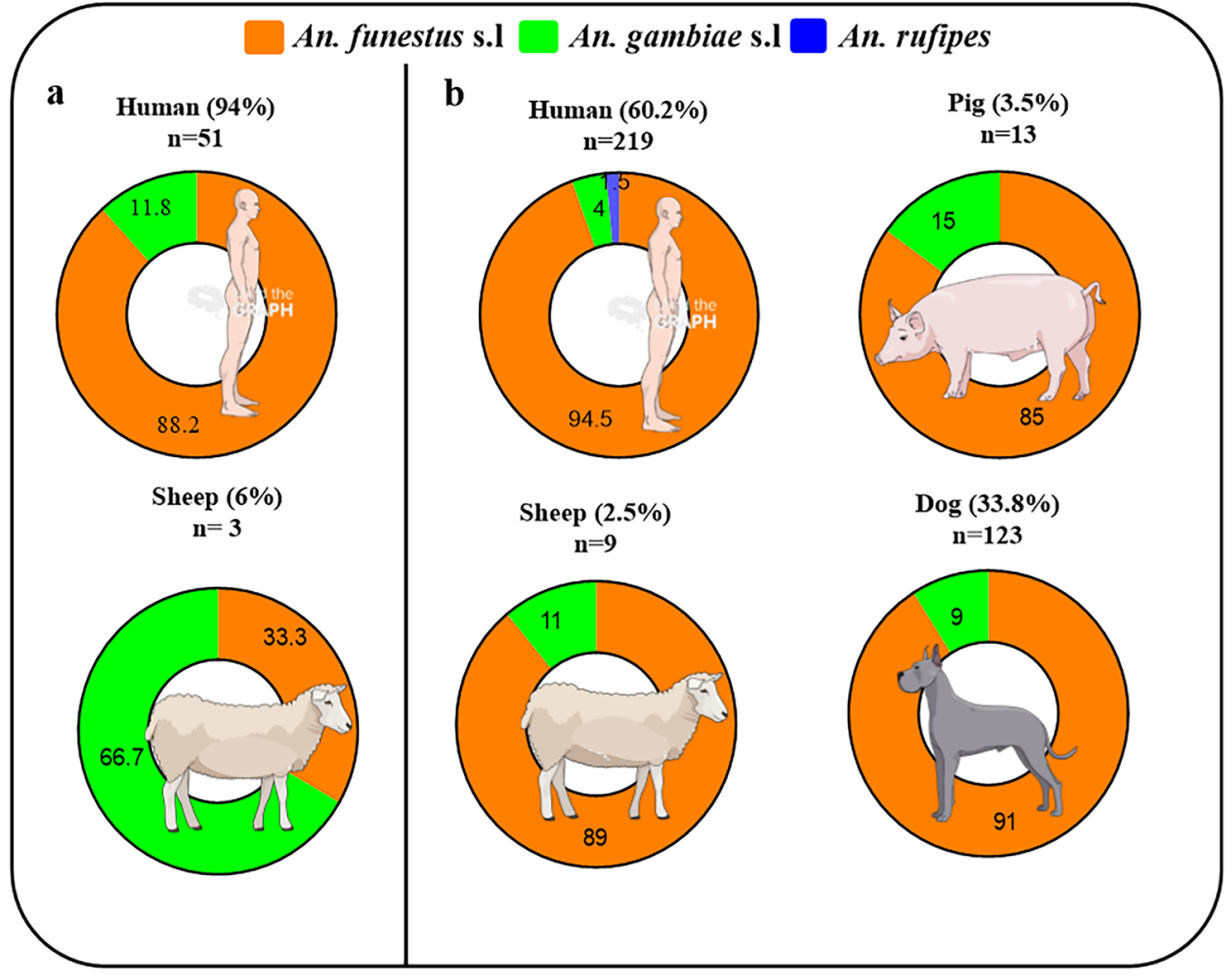
Influence of L119F GSTe2 metabolic resistance on some malariometric indices in Elende and Mibellon. **a & c**: Association between L119F-Gste2 genotypes and *Anopheles funestus*’s biting behavior, Parity status, sporozoite infection and biting pattern in Elende; **b & d**: Association between L119F-Gste2 genotypes and *An. funestus*’ biting behavior, Parity status, sporozoite infection and biting pattern in Mibellon. **RR**: 119 F/F homozygous resistant; **RS**: L119F heterozygous; **SS**: L/L119 homozygous susceptible

In Elende, the 69% (399/579) of parous mosquitoes were comprised of 35% (140/399) 119 F/F-RR homozygous resistant, 42% (168/399) L119F-RS heterozygous, and 23% (91/399) L/L119-SS homozygous susceptible, with no significant difference observed in the distribution of the three genotypes in nulliparous mosquitoes (χ^2^ = 1.6, p = 0.4). The same observation was made in Mibellon (χ^2^ = 0.1, p = 0.9) (Fig. 8a & b). However, in Mibellon, 119 F/F-RR homozygous resistant vectors showed a tendency to be more nulliparous, whereas the L/L119-SS homozygous susceptible were more parous (Fig. 8b). The same trend was observed in Elende only for 119 F/F-RR mosquitoes (Fig. 8a).

#### Association between the L119F-*GSTe2* resistant marker and *An. funestus’* biting behaviour and aggressivity

In Elende, the 48% (837/1744) of mosquitoes collected outdoors were comprised of 35% (297/837) 119 F/F-RR homozygous resistant, 41% (345/837) L119F-RS heterozygous, and 24% (195/837) L/L119-SS homozygous susceptible. A similar distribution of the three genotypes was observed for mosquitoes collected indoor showing no significant difference (χ^2^ = 0.08, p = 0.9) in biting preference (Fig. 8a). In Mibellon, a similar trend was observed with no significant association between L119F-*GSTe2* genotypes and the biting behaviour (χ^2^ = 0.3, p = 0.86) although 119 F/F-RR homozygous resistant individuals tended to bite more outdoor than indoor (Fig. 8b).

Irrespective to the site, the aggressivity of mosquitoes with all the three genotypes started very early in the evening (06:00 p.m) till late in the morning, with a higher proportion of 119 F/F-RR and L119F-RS mosquitoes recorded at 07:00 a.m compared to the L/L119-SS mosquitoes (Fig. 8c & d). The RR and RS individuals had a similar peak biting time around 04–05 a.m. in both locations whereas it was between 02 and 04 am for the SS mosquitoes.

#### Association between the L119F-GSTe2 resistant marker and *An. funestus’* infectivity and Entomological inoculation rate (EIR)

Regarding the infectious status, in Elende, the 8.7% (66/755) of infected mosquitoes included 40% (26/66) 119 F/F-RR homozygous resistant, 42% (28/66) L119F-RS heterozygous and only 18% (12/66) L/L119-SS homozygous susceptible. As observed with the two first entomological indices, similar distribution of the three genotypes was observed with the opposite index (non-infected status) (χ^2^ = 0.8, p = 0.6). In Mibellon, there was also no significant association between L119F-GSTe2 genotypes and infectious status (χ^2^ = 0.3, p = 0.86). Surprisingly, whatever the site, 119 F/F-RR homozygous resistant vectors showed a tendency to be more infected (Fig. 8a & b), whereas the L/L119-SS homozygous susceptible were more often non-infected in Elende (Fig. 8a).

Analysis of the association between L119F-*GSTe2* and Entomological Inoculation Rate (EIR) was performed only in Elende because of the high frequency of the 119 F-*GSTe2* resistant allele in this location enabling a better comparison. In this location, monthly malaria transmission intensities associated to the 119 F/F-RR (6.6 ib/h/m) and L119F-RS (7.5 ib/h/m) mosquitoes were significantly higher compared to that of L/L119-SS mosquitoes (3 ib/h/m) (IRR = 2.2, 95%CI (1.1–5.2), p = 0.03; IRR = 2.5, 95%CI (1.2–5.8), p = 0.01 respectively). However, there was no significant difference between 119 F/F-RR and L119F-RS mosquitoes (IRR = 0.8, 95%CI (0.5–1.6), p = 0.6) (Table 4).

**Table 4:** Influence of L119F GSTe2 genotypes on malaria transmission intensity in Elende

Sites	Entomological parameters	L119F GSTe2 genotypes (HLC)			Global	Sites	Statistical analysis		
							L119F GSTe2 genotypes	Entomological Inoculation Rate (EIR)	
		RR	RS	SS				IRR (95%CI)	p-value
Elende	SIR (%)	9.4	9.1	6.9	8.7				
	HBR	2.3	2.7	1.5	6.5		RR vs RS	0.8 (0.5–1.6)	0.6
	EIR (b/h/n)	0.22	0.25	0.1	0.57	**Elende**	RR vs SS	2.2 (1.1–5.2)	0.03^*^
	MEIR (ib/h/m)	6.6	7.5	3	17.1		RS vs SS	2.5 (1.2–5.8)	0.01^*^

## Discussion

The present study aimed to investigate the impact of GST-based metabolic resistance on the vectorial capacity of the major malaria vector *An. funestus* through a longitudinal study. The study highlighted the predominant role of *An. funestus* in the studied locations, a high level of malaria transmission and also revealed that GST-based resistance is increasing malaria transmission.

### *An. funestus* was perennially the predominant species in the two collection sites

*Anopheles* mosquitoes collected by HLC from both sites were almost exclusively made up of *An. funestus* s.l. following by *An. gambiae* s.l confirming previous reports [17, 18]. High abundance of *An. funestus* s.l was reported in other locations in Cameroon [17, 26, 29], in Kenya [30], Tanzania [31] and could be linked to the presence of permanent large pools of water in both sites known to be their favourite breeding site. The high predominance of *An. funestus* s.s as shown by molecular identification is similar to the previous studies carried out in the same collection sites [18, 26], and in Tibati and Gounougou (Northern Region) [14, 32]. The dominance of *An. funestus s.s* in those localities further confirms the extremely high anthropophilic status of this species and its strong potential involvement in disease transmission. With regard to the *An. gambiae* complex, in Elende, the presence of both *An. gambiae* s.s. (85%) and *An. coluzzii* (15%) was consistent with the standard pattern which shows that both species are widely distributed in the country [32, 33]. The presence of *An. arabiensis* (4%) in Mibellon (Adamawa Region) contrasts the general trend showing so far that this species was restricted to the Northern arid and semi-arid zone of the country [32, 33]. The presence of *An. nili* s.s as the sole species of the group is similar to the studies conducted in Nyabessang (Southern Region) [34], in the Northern part of the Benin [35], in the Southern and Western forest areas of Côte d’Ivoire [36] and in the Sudano-Guinean Zone of Senegal [37]. Secondary malaria vectors such as *An. ziemanni*, *An. nili* s.l and *An. rufipes* which are better-known as zoophilic species [38, 39] collected in these sites indicate high vector diversity and potential increase risk of malaria transmission.

### Entomological indices recorded support a high malaria transmission level in both location

*An. funestus* s.l, *An. gambiae* s.l and *An. nili* s.l all had very high parity rates ranging from 52 to 100%, showing that vectors lived long enough to complete the sporogony cycle of the parasite to become infectious. Similar observations were highlighted by Fondjo et al. [34] in different localities of the country. This could indicate a limited efficacy of vector control tools in these locations potentially associated with escalation of pyrethroid resistance [40].

Malaria transmission dynamic is highly influenced by host preference and feeding behaviour of mosquito vectors. The very high overall HBI (single + mixed) of 94% in each site highlights the strong anthropophagic tendency of the main malaria vectors *An. funestus* s.s. and *An. gambiae* s.s. in Cameroon and corroborates with previous reports of Antonio-Nkondjio et al. [41], Tanga et al. [42] and Tabue et al. [39] in the Central, South-west and Northern part of the country. Surprisingly, *An. rufipes*, previously known as a zoophilic species [19], was found in this study as the only species taking the blood meal solely on humans. The small sample size of *An. rufipes* in this study could be a limitation of the observed result, therefore no strong conclusions could be drawn. However, studies conducted in the Northern part of the country showed the highly opportunistic role of this species regarding its feeding behaviour associated with a pronounced zoophilic tendency [43, 44]. Considering the fact that its epidemiological role in malaria transmission has been recently highlighted in the country [39, 43], our results suggest that routine entomological surveillance in the area where this species is most abundant should be carried out to update its vectorial capacity. The relatively high feeding on Dog (Index = 53%) observed in Mibellon, suggests that use of insecticide-treated dogs could be a control approach in such areas as other authors have done previously using cattle [45]. The proportion of mixed blood meals was higher than that of single blood, with *An. gambiae* s.l. and *An. funestus* s.s. feeding on several hosts. It is becoming recurrent to find species of the *An. gambiae* complex and the *An. funestus* group with human and animal blood in Cameroon [39, 44] or in other tropical African countries [46, 47]. Finally, our results also showed an opportunistic behaviour of these species in terms of their propensity to feed on four different hosts (human, dog, pig and sheep). This multiple feeding could be attributed to the low nutritional content of the first meal ingested, defensive behaviour or host movement, and environmental factors such as wind movement [48].

*Anopheles* species from both sites were aggressive throughout the night both indoors and outdoors, with biting activity noticeably starting early in the evening (6p.m) and ending late in the morning, up to 8a.m in the specific case of *An. funestus* s.l. This early and late biting activities of *Anopheles* mosquitoes which is consistent with human behaviour, correlates with findings from different parts of the country [32, 49] and other tropical African countries [49–51] and may be due to the use of insecticide-treated bednets, which may induce mosquitoes to blood-feed at places and at times when humans are not protected. This result also suggests that studies are needed to investigate a possible daytime malaria as recently reported in Central African Republic (Bangui) where 20–30% of indoor bites occurred during the day and the prevalence of *Plasmodium falciparum* in night and day biting mosquitoes was the same [51]. The overall hourly activity of *An. funestus* s.l and *An. gambiae* s.l was significantly higher in the second part of the night (00–08 a.m) in both sites when people are supposed to be asleep. The Human Biting Rate (HBR) of *An. funestus* s.l. was significantly higher indoor than outdoor at both sites confirming its high endophagic tendency.

### *An. funestus* sustains a very high entomological inoculation rate (EIR) in both locations

This study revealed a very high *Plasmodium* sporozoite infection rate across the two sites in *An. funestus* s.l mosquitoes. This infection rate was similar to some reports across Cameroon in the same mosquito species by Nkemngo et al. [26], Ndo et al. [52], Mieguim et al. [53] and was higher than recent reports across Africa by Osse et al. [35] in Benin, Mapua et al. [31] in Tanzania, Wolie et al. [54] in Côte D’Ivoire and Bamou et al. [49] in Kenya and in Ethiopia. The variations between these rates could be attributed to the differences in the detection methods used (TaqMan, ELISA and Nested-PCR). *An. funestus* s.l and *An. gambiae* s.l, were the only species found infected, confirming their key role as major malaria vectors and in Cameroon [17, 26]. Among *Plasmodium* species infecting the salivary glands of *An. funestus* s.l mosquitoes, *P. falciparum* was by far the predominant species counting for 92 and 70% of the global infection respectively in Elende and Mibellon. This result corroborates recent reports carried-out by Nkemngo et al. [26], Tchouakui et al. [29], Mbama-Ntabi et al. [55] (using human blood) and give right to the importance that malaria control strategies attributes against this species. The high proportion of *P. malariae* in Mibellon suggests that control programs should also pay attention to secondary parasite *P. malariae* and *P. ovale* in the prospect of malaria elimination efforts.

*An. funestus* populations from forested area (Elende) exhibited the same malaria transmission intensity than those from the humid savannah (Mibellon) with 66 ib/h/m (792 ib/p/year) on each site. This malaria transmission intensity represents one of the highest across the continent [4, 31, 35, 42, 49, 53] highlighting the high transmission of malaria in Cameroon, which is still part of the 11 countries with the highest burden in the world. This high implication of *An. funestus* s.l mosquitoes in malaria transmission corroborates with results obtained recently in Tanzania by Mapua et al. [31] and Kaindoa et al. [56] where *An. funestus* contributed for 97.7 and 86.26% of all malaria transmission respectively. This could be strongly correlated to the high affinity of *An. funestus* s.l mosquitoes to humans over other vertebrates as demonstrated previously by the very high HBI; their high resistance to pyrethroid insecticides used in LLINs which allow them to live much longer and become more infected [57–59], and their preference for permanent and semi-permanent aquatic habitats which last far longer than the rainy season [31]. Hence, our results suggest that persistent residual transmission is strongly associated with *An. funestus* s.l implying that targeted, high-impact, species-specific interventions could improve control of this vector. This further shows that the residual malaria transmission is not only related to zoophilic “secondary” vectors [51, 60], but often involves the “usual vector suspects” exhibiting opportunistic feeding and resting behaviors [61]. This opportunistic feeding and resting behaviors of ‘’usual vector suspects’’ is further confirmed by the very high malaria transmission intensity observed outdoor for *An. funestus* s.l mosquitoes (48 and 39 ib/h/m (576 and 468ib/h/y) in Mibellon and Elende respectively), supported by the fact that in Elende, transmission intensity was significantly equal indoor and outdoor. In the context of malaria elimination in the coming years, this latter observation illustrates the imperative of designing externally based vector control interventions complementary to the in-house interventions (LLINs, IRS).

### Non-uniform distribution of L119F-*GSTe2* resistance allele and high biting pattern of 119 F/F resistant *An. funestus* s.s at dawn

The L119F mutation was detected in both localities with a significantly higher frequency of the resistant allele in Elende (56%). The frequency of the resistant allele observed in Mibellon (35%) is almost similar to those obtained at the same site 3–4 years ago by Ndo et al. [62] (29.95%) and Tchouakui et al. [29] (26.3%), the slight differences observed being attributed to the study protocol used, the latter authors not having conducted longitudinal surveys and used only indoor resting female mosquitoes. This difference in terms of 119 F resistance allele frequency at both sites could be attributed to different insecticides selection pressures.

The biting pattern of 119 F/F-RR homozygous resistant mosquitoes showed that the 04–05 a.m. time slot represents their prime activity range at both sites. The result is quite similar with the one of Githinji et al. (2020) [63] in the western Kenya which showed that the 03–04 a.m was the prime activity range of kdr resistant *An. gambiae* mosquitoes. Together with a high proportion of mosquitoes carrying this genotype biting after 6 a.m. compared to other genotypes, these observations imply a high activity of 119 F/F resistant mosquitoes at dawn, especially when people are likely to be awake and out of their chemical or physical lethal barrier (LLIN). This result may be explained by the fact that the increased expression of *GSTe2* in these mosquitoes helps them to avoid exposure at least partially to insecticide-treated nets during the hours when people are asleep and protected. This observation is in accordance with Menze et al. [57] during an experimental hut evaluation of treated nets against *An. funestus* s.s, where they showed that mosquitoes carrying the L119F-*GSTe2* mutation were significantly associated with increased exophily to Olyset Plus and PermaNet 2.0 nets. This change in the biting pattern of 119 F/F resistant *An. funestus* s.s mosquitoes, with a heavy biting at dawn, could also explain residual malaria transmission.

### The L119F-*GSTe2* metabolic gene is associated with the high malaria transmission intensity

This assessment of the impact of L119F-*GSTe2* metabolic gene on the malaria transmission intensity in Africa revealed that the L119F-*GSTe2* mutation was strongly associated with increased EIR mainly by increasing *Plasmodium* infection rate in Elende. In fact, mosquitoes bearing 119 F-*GSTe2* resistant allele (RR and RS) were significantly more likely to transmit malaria compared to their susceptible counterparts (SS). Similar observation showing that the L119F-*GSTe2* metabolic gene increases *An. funestus* vector competence was reported in the central part of the country by Tchouakui et al. (2019) [14] and Ndo et al. (2019) [62] and could have four main explanations. Firstly, the resistance status conferred by the L119F-*GSTe2* gene could explain this phenomenon, as mosquitoes carrying the resistant 119 F-*GSTe2* allele live longer due to their ability to tolerate the effects of insecticide exposure in the field, and subsequently being more likely to enable parasites to complete their extrinsic incubation period than homozygous susceptible mosquitoes [29]. The second explanation is that, the increased susceptibility of mosquitoes to infection may be related to the overproduction of GSTs that could protect the parasites from the effects of reactive oxygen species (ROS) known to play a role in the innate immune responses of insects as a potent pathogen-killing agent [64]. The third is that resource-based trade-offs could have affected mosquito immune-competency in such a way that, overproduction of detoxifying enzymes such as GSTs is likely to deplete the resource pool, limiting the vector’s ability to mount a strong immune response, therefore favouring the development of the parasite [64]. The fourth is related to the change of the biting pattern of L119F-*GSTe2* resistant *An. funestus* s.s mosquitoes as mentioned above, leading to an increased biting at times when peoples are likely to be awake and out of their chemical or physical lethal barrier (LLINs) as compared to the susceptible mosquitoes. The fact that L119F- *GSTe2* resistance marker increases malaria transmission intensity is of great concern for the epidemiology of malaria in sub-Saharan Africa and in Cameroon in particular, further explaining the high epidemiological role of *An. funestus* s.s and the priority to also design species-specific interventions. This result is in agreement with the fact that the continuous increase of insecticide resistance under field conditions has a serious impact on malaria transmission, in particular, when this resistance is mediated by resistance markers like L119F-*GSTe2* known to affect vector longevity [65, 66]. Owing to the fact that mosquitoes sometimes wear more than one insecticide resistance markers, additional work need to be done to further assess the combined effect of insecticide resistance marker on EIR across the continent.

## Conclusion

This study reveals a predominance of *An. funestus* s.l. sustaining a high and perennial malaria transmission intensity both indoor and outdoor in two locations in Cameroon. Furthermore, the L119F-*GSTe2* metabolic gene, one of the main resistance markers in *An. funestus* s.s., was significantly associated with an increase of malaria transmission intensity in this species. This shows that the GST-based metabolic resistance potentially increases malaria transmission in resistant *An. funestus* mosquitoes which could lead to an exacerbation of malaria transmission in areas with high *GSTe2*-based metabolic resistance to insecticides.

### Electronic supplementary material

Below is the link to the electronic supplementary material.

Supplementary Material 1

## Data Availability

The datasets generated and/or analysed during the current study are presented in the manuscript and in Additional file 1 openly available in figshare at 10.6084/m9.figshare.23297975.

## List of abbreviations

PCR: Polymerase Chain Reaction
AS-PCR: Allele Specific PCR
ELISA: Enzyme-Linked Immuno-sorbent Assay
HLC: Human Landing Catch
CDC-LT: Centre for Diseases Control Light Trap
PSC: Pyrethrum Spray Catch
MHBR: Monthly Human Biting Rate
SIR: Sporozoite Infection Rate
EIR: Entomological Inoculation Rate
ib/h: infected bites / human
HBI: Human Blood Index
LLINs: Long Lasting Insecticide Nets
IR: Insecticide Resistance
*GSTe2*: Glutathione S-Transferase epsilon 2
*CYP450*: Cytochrome P450
RR: Homozygous Resistant
RS: Heterozygous
SS: Homozygous Susceptible
