# Supplementary material for: Entomological longitudinal surveys in two contrasted eco-climatic settings in Cameroon reveal a high malaria transmission from Anopheles funestus associated with GSTe2 metabolic resistance
Source: BMC Infect Dis. 2023 Oct 28;23:738. doi: 10.1186/s12879-023-08698-8 (PMC10612181; doi:10.1186/s12879-023-08698-8)
Supplement: Supplementary file 1 — Supplementary Material 1 [file 12879_2023_8698_MOESM1_ESM.docx]

Additional file 1

Tables

**Table S1**: How to compute entomological parameters

| **Entomological parameters** | **How to compute** |
| --- | --- |
| Human Blood Index (HBI) or Dog Blood Index (DBI) | [Total number of positive in ELISA for human blood (or Dog blood)/total number of tested mosquitoes] ×100 |
| Human Biting Rate (HBR) | Total number of collected mosquitoes/ (total number of volunteers x number of collection nights) |
| Monthly Human Biting Rate (MHBR) | [Total number of collected mosquitoes/ (total number of volunteers x number of collection nights)]x30 |
| Sporozoite Infection Rate (SIR) | (Total positive with Nested PCR/total tested) ×100 |
| Entomological Inoculation Rate (EIR) | SIR x HBR |
| Monthly Entomological Inoculation Rate (MEIR) | (SIR x HBR) x30 |

**Table S2**: Anopheline mosquito’s parity rate in Elende and Mibellon.

| **Species** | **Sentinel sites** | **Total dissected (N)** | **Parous n (%)** | **IRR (95% IC)** | **p-value** |
| --- | --- | --- | --- | --- | --- |
| ***An. funestus* s.l** | Elende | 898 | 552 (61.5) | 1.2 (1.02-1.3) | 0.02 |
|  | Mibellon | 799 | 564 (70.6) |  |  |
| ***An. gambiae* s.l** | Elende | 161 | 108 (67) | / | / |
|  | Mibellon | / | / |  |  |
| ***An. nili* s.l** | Elende | 87 | 61 (70) | / | / |
|  | Mibellon | / | / |  |  |
| **Total** |  | **1945** | **1285 (66.1)** |  |  |
| ***Anopheles* sp.** | Elende | 1146 | 721 (63) | 1.1 (1.01-1.2) | 0.04 |
|  | Mibellon | 799 | 564 (70.6) |  |  |

**Table S3**: Single and mixed Blood meal of wild caught *Anopheles* mosquitoes from Elende and Mibellon

| **Sites** | **Species** | **Total tested (Positive and Negative)** | **Single Blood Meal** | | **Mixed Blood Meals** | | | | |
| --- | --- | --- | --- | --- | --- | --- | --- | --- | --- |
|  |  |  |  |  | **Mixed (n=2)** | | **Mixed (n=3)** | | **Mixed (n=4)** |
|  |  |  | **Human**  **N (%)** | **Sheep**  **N (%)** | **Human+Dog**  **N (%)** | **Human+Sheep N (%)** | **Human+Sheep+Dog**  **N (%)** | **Human+Pig+Dog**  **N (%)** | **Human+Sheep+Pig+Dog**  **N (%)** |
| **Mibellon** | *An. funestus* s.s | 212 | 92 (43) | 0 (0) | 107 (51) | 1 (0.5) | 1 (0.5) | 5 (2) | 6 (3) |
|  | *An. gambiae* s.l | 18 | 9 (50) | 0 (0) | 7 (38) | 0 (0) | 0 (0) | 1 (6) | 1 (6) |
|  | *An. rufipes* | 3 | 3 (100) | 0 (0) | 0 (0) | 0 (0) | 0 (0) | 0 (0) | 0 (0) |
|  | **Total tested** | **233** | **104 (45)** | **0 (0)** | **114 (49)** | **1 (0.5)** | **1 (0.5)** | **6 (2)** | **7 (3)** |
| **Elende** | *An. funestus* s.s | 46 | 45 (98) | 1 (2) | 0 (0) | 0 (0) | 0 (0) | 0 (0) | 0 (0) |
|  | *An. gambiae* s.l | 8 | 6 (75) | 2 (25) | 0 (0) | 0 (0) | 0 (0) | 0 (0) | 0 (0) |
|  | **Total tested** | **54** | **51 (94)** | **3 (6)** | **0 (0)** | **0 (0)** | **0 (0)** | **0 (0)** | **0 (0)** |

n=Vertebrate host number; N=Mosquito number found with vertebrate host antigen(s)

**Table S4**: Entomological indices of *Anopheles funestus* s.l. mosquitoes collected per month by HLC in Elende.and Mibellon

| Sites | Collection Months |  | Biting Place | | | | | | Overall collection | | | | |
| --- | --- | --- | --- | --- | --- | --- | --- | --- | --- | --- | --- | --- | --- |
| Elende |  | Indoor | | | | Outdoor | | | Indoor+Outdoor | | | | |
|  |  | SIR (%) 95%CI (Tested) | | HBR (b/h/n) | EIR (ib/h/n) | SIR (%) 95%CI (Tested) | HBR (b/h/n) | EIR (ib/h/n) | SIR (%) 95%CI (Tested) | HBR (b/h/n) | EIR (ib/h/n) | MHBR (b/h/m) | MEIR (ib/h/m) |
|  | Dec.19 | 5.8 [0.7-21] (36) | | 19.5 | 1.1 | 3.3 [0.8-18] (30) | 12.4 | 0.4 | 4.7 [1-13] (66) | 16 | 0.7 | 480 | 21 |
|  | Jan. 20 | 8.5 [0.3-20] (59) | | 53 | 4.5 | 0 [0] (62) | 44 | 0 | 4.1 [1-9] (121) | 48.5 | 2 | 1455 | 60 |
|  | Feb. 20 | 2 [0.5-11] (50) | | 58.5 | 1.2 | 0 [0] (37) | 39.5 | 0 | 1.2 [0.03-6] (87) | 49 | 0.6 | 1470 | 18 |
|  | Mar. 20 | 8.5 [0.2-22] (47) | | 22.3 | 2 | 23 [13-38] (65) | 14.7 | 3.4 | 17 [10-26] (112) | 18.5 | 3.1 | 555 | 93 |
|  | Jul. 20 | 31 [18-49] (55) | | 17.8 | 5.5 | 4.3 [0.1-24] (23) | 10.5 | 0.5 | 23 [14-36] (78) | 14.2 | 3.3 | 426 | 99 |
|  | Aug. 20 | 7 [2-18] (57) | | 27.7 | 2 | 6.7 [0.8-24] (30) | 17 | 1.1 | 6.9 [2.5-15] (87) | 22.4 | 1.5 | 672 | 45 |
|  | Sep. 20 | 10.3 [4-22] (58) | | 27.7 | 2.8 | 0 [0] (14) | 17.2 | 0 | 8.3 [3-18] (72) | 22.5 | 2 | 675 | 60 |
|  | Oct. 20 | 12.2 [4-28] (41) | | 27.4 | 3.3 | 0 [0] (24) | 15 | 0 | 7.7 [2.5-18] (65) | 21.2 | 1.6 | 636 | 48 |
|  | Nov. 20 | 4.4 [0.5-16] (45) | | 13.6 | 0.6 | 4.2 [0.1-23] (24) | 9.5 | 0.4 | 4.3 [0.8-13] (69) | 11.5 | 0.5 | 345 | 15 |
|  | **Total** | **10.3 [7-14] (446)** | | **29.7** | **3.1** | **6.5 [4-10] (309)** | **20** | **1.3** | **8.7 [6.7-11] (755)** | **25** | **2.2** | **750** | **66** |
| Mibellon | Jan.21 | 7.7 [3.5-14.6] (117) | | 17.6 | 1.3 | 1.8 [0.2-6.6] (109) | 11.5 | 0.207 | 5 [2.4-8.7] (226) | 14.5 | 0.7 | 435 | 21 |
|  | Mar.21 | 9.2 [5.2-15] (174) | | 16.1 | 1.5 | 4.5 [1.4-10] (112) | 11.8 | 0.531 | 7.3 [4.5-11] (286) | 14 | 1 | 420 | 30 |
|  | May.21 | 6.7 [3-12.7] (134) | | 15.5 | 1.04 | 8.2 [3.6-16] (97) | 8.4 | 0.6888 | 7.4 [4.2-12] (231) | 12 | 0.8 | 360 | 24 |
|  | Jul.21 | 6.6 [3.4-11.6] (181) | | 16 | 1.06 | 13.1[8.4-19.3] (191) | 14.6 | 1.9126 | 10 [7-13.7] (372) | 15.3 | 1.5 | 459 | 45 |
|  | Sep. 21 | 33 [23-45] (115) | | 40.3 | 13.3 | 7.7 [3.5-14.7] (116) | 42.3 | 3.2571 | 20.3 [15-27] (231) | 41.3 | 8.4 | 1239 | 252 |
|  | Nov. 21 | 20.5 [13-30] (117) | | 22.1 | 4.5 | 11.4 [6-19.5] (114) | 25.1 | 2.8614 | 16 [11.2-22] (231) | 23.6 | 3.8 | 708 | 114 |
|  | **Total** | **12.8 [10.5-15.5] (838)** | | **21.3** | **2.7** | **8.4 [6.4-10.7] (739)** | **19** | **1.596** | **11 [9.2-12.5] (1577)** | **20.2** | **2.2** | **606** | **66** |

b/h/n: bites/human/night; b/h/m: bites/human/month; ib/h/n: infected bite/human/night; ib/h/m: infected bite/human/month
